# A new fat-dissociation method to detect lymph nodes in colorectal cancer: a prospective randomized study

**DOI:** 10.1038/s41598-020-77195-8

**Published:** 2020-11-19

**Authors:** Shiki Fujino, Norikatsu Miyoshi, Masayuki Ohue, Aya Ito, Masayoshi Yasui, Takayuki Ogino, Hidekazu Takahashi, Mamoru Uemura, Chu Matsuda, Hirofumi Yamamoto, Tsunekazu Mizushima, Yuichiro Doki, Hidetoshi Eguchi, Nariaki Matsuura

**Affiliations:** 1 Innovative Oncology Research and Translational Medicine, Osaka International Cancer Institute, 3-1-69, Otemae, Chuo-ku, Osaka, 541-8567 Japan; 2 Department of Gastroenterological Surgery, Osaka University Graduate School of Medicine, 2-2, Yamadaoka, Suita, Osaka 565-0871 Japan; 3 Department of Surgery, Osaka International Cancer Institute, 3-1-69, Otemae, Chuo-ku, Osaka, 541-8567 Japan

**Keywords:** Gastroenterology, Medical research, Translational research, Oncology, Cancer, Surgical oncology

## Abstract

Histological examination of the lymph nodes (LNs) is crucial to determine the colorectal cancer (CRC) stage. We previously reported a new fat-dissociation method (FM) to detect LNs from surgically resected mesentery. This study aimed to examine the effectiveness of FM compared with that of conventional palpation method (PM) in CRC. This single-center, open-label, randomized controlled study was performed at Osaka International Cancer Institute in Japan in 2014. Randomization was performed using a computer-generated permuted-block sequence. Patients were stratified by surgical procedures and the LN dissection area. The primary endpoint was the time required for LN identification. The secondary endpoint was the number of LNs and 5-year cancer-specific survival. The 130 enrolled patients were randomly assigned in a 1:1 ratio to the FM and the PM groups. LN identification times were 45 (range 15–80) and 15 (range 7–30) minutes in the PM and the FM groups, respectively (*P* < 0.001). In the PM group, body mass index and identification time were correlated (*P* = 0.047). The number of LN which could be examined pathologically was 16 (range 2–48) and 18 (range 4–95) in the PM and FM groups, respectively (*P* = 0.546). In right-sided CRC, the number of LNs was higher in the FM group than in the PM group (*P* = 0.031). Relapse-free survival rates and cancer-specific survival rates did not differ between the groups. In conclusion, FM reduced the time required for LN detection without reducing the number of detected LNs, making it is a useful method to detect LNs in surgical specimens.

## Introduction

Cancer is a leading cause of death worldwide, with approximately 17 million individuals dying of cancer annually^1^, and the incidence and mortality rates of cancer are still projected to increase. Colon, lung, breast, liver, and stomach cancers account for the majority of cancer-related deaths. Specifically, colorectal cancer (CRC) is the second most frequent cancer in Europe^2^ and the second most common cause of death in the US^3^. Also, in Asian countries, CRC is the leading cause of death with the number of cases identified continuing to increase^4^. In CRC treatment, surgical resection of the primary tumor and regional lymph nodes (LNs) is very important^5^, and TNM classification is determined by histological examination of a surgically resected specimen. Surgical specimens with proximal, distal, and circumferential margins, regional LNs, tumor classification, and vascular invasion are usually examined. The T factor depends on tumor invasion depth, while the N factor depends on the number of LN metastases. It is especially recommended to examine at least 12 LNs because the small number of LNs examined results in downstaging because of missing LN metastases^6–9^. Furthermore, the adjuvant chemotherapy regimen is defined based on the number of LN metastases^10^.

The detection method for LNs varies from country to country. In Japan, surgeons isolate LNs directly from the surgically resected specimen and then fix them in formalin. This procedure requires time and expertise for recognizing LNs from surgically resected specimens. In other countries, the surgical specimen is first fixed in formalin and then examined using the cross-section of the tissue or after fat clearance^11,12^ using Schwartz solution^13^ or GEWF solution^14,15^, or after the pathologist isolates the lymph nodes. Both solutions reduced the fat volume of the specimen to reveal LNs; however, these methods are time consuming and require from 1 to 9 days to complete. Therefore, improved methods are required for more efficient LN identification. Recently, we reported the importance of micro-metastasis as a valuable prognostic marker^16^. Concurrently, one-step nucleic acid amplification (OSNA) has been introduced into clinical use for the examination of micro-metastasis^17,18^. This method has the advantage of high precision of micrometastases, and has performed well in searching for micrometastases of sentinel lymph node in various tumors^19–21^. However, for the successful use of OSNA, it is necessary to isolate LNs directly from the specimen. The conventional method for LN isolation is the palpation method (PM). However, PM is very time consuming and depends on the experience of the physician, and in Japan, many of the physicians performing PM are surgeons. Because of the limitations associated with current methods, a universal method that is less time consuming and does not require extensive experience is needed. We previously reported a new effective method called the fat-dissociation method (FM) for LN detection^22^. This method enabled the dissociation of mesenteric fat and clear visualization of the LNs, making LN detection directly from the specimen easier. This new FM could be performed within 1 h, and it did not affect the histological examination.

The present study aimed to examine the clinical usefulness of FM with respect to LN detection time and the number of LNs. We hypothesized that the new FM is superior to the conventional PM method. Towards our goal, we evaluated the differences in time required for detecting LNs from surgically resected specimen and in the total number of detected LNs between the two methods.

## Material and methods

### Study design and participants

This was a single-center, open-label, randomized controlled study conducted at Osaka International Cancer Institute (OICI) in Japan from January to December 2014. The OICI ethics committee approved this study, and written informed consent was obtained from all patients. This study was performed in accordance to guidelines of Declaration of Helsinki. Patients who underwent surgery of primary CRC with dissection of LNs and aged 20 years or older were eligible. For rectal cancer, those whose tumor location was in the lower rectum below peritoneal reflection were excluded because given the several operative approaches^23^. Those whose tumor location was in the upper rectum above peritoneal reflection were included because the surgical procedure was anterior resection or low anterior resection. The enrolled patients were randomized in a 1:1 ratio for use of either the PM or the FM for detecting LNs from surgically resected specimen. Randomization was conducted using a computer-generated permuted-block sequence. The size of the blocks used for randomization was set to two. Patients were randomly assigned and balanced according to surgical procedure (ileocecal resection, right colectomy, transverse colectomy, left colectomy, sigmoid colectomy, anterior resection, and low anterior resection) and the dissection area of LNs (D2 or D3 dissection). The defined LN dissection was performed for each CRC location according to the Japanese Classification of Colorectal Carcinoma guidelines^23^.

### Sample size

The sample size was calculated using R 3.1.3 software (CRAN, the R Foundation for Statistical Computing, Vienna, Austria). We examined 10 patients retrospectively prior to the start of this study and the time for LN detection was shorter by 30% in the FM group (data not shown). A sample size of 59 assessable patients in each group was required to achieve 90% power with 5% error (two-sided). A total of 118 patients was necessary in this study. From the number of the CRC surgery of our institute, we scheduled a 1-year study.

### Outcomes and assessments

The primary efficacy endpoint was the time required to identify LNs. The secondary endpoint was the number of LNs and 5-year cancer-specific survival (CSS). After surgery, follow-up included assessments of serum concentrations of the tumor markers, carcinoembryonic antigen and carbohydrate antigen 19-9. Further examinations included imaging with abdominal ultrasonography, computed tomography, and/or positron emission tomography was conducted every 3–6 months and colonoscopy in accordance with Japanese guidelines^8^. Relapse-free survival (RFS) was defined as the length of time that the patients survived without any signs or symptoms of cancer after primary colorectal cancer surgery. CSS was defined as the length of time after primary colorectal cancer surgery that the patients survived.

### PM and FM for LN detection

**Figure 1:**
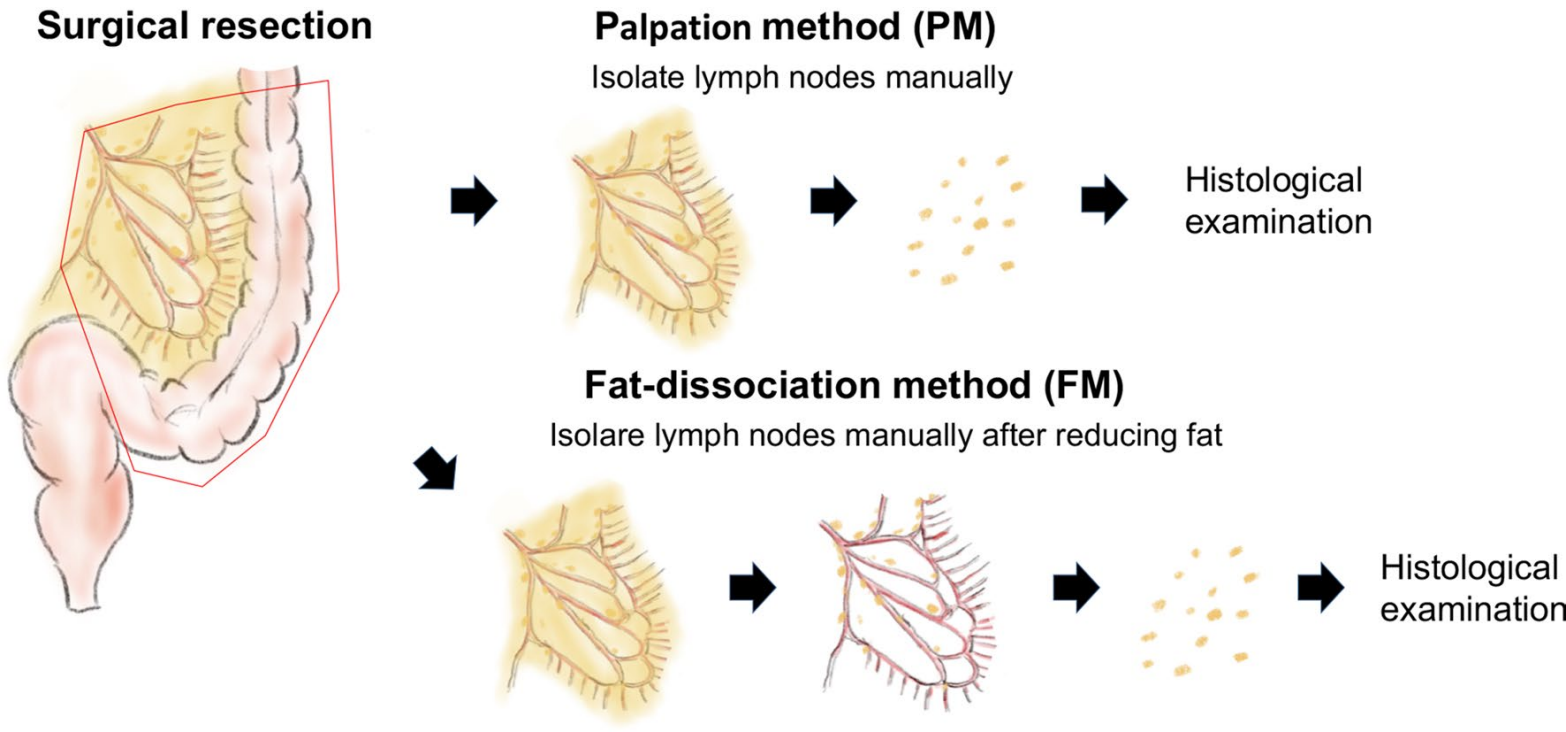
Schematic for the fat-dissociation method. The mesentery was separated from the primary tumor lesion, and the fat-dissociation solution was added. For FM, after about 30–60 min of incubation in FD solution at 40 °C, the mesentery was taken out of the solution and the lymph nodes were isolated.

The clinical tissue samples were obtained by surgery of the primary CRC. The mesentery was carefully separated from the primary tumor lesion (Fig. 1). In the PM group, gastrointestinal surgeons isolated LNs by palpating the specimen manually. In the FM group, the mesenteric tissue was dissociated by using the fat-dissociation solution^22^. Next, 1 ml of the solution was added to 3 g of mesentery specimen. The samples were incubated at 40 °C between 30 and 60 min. Next, the mesentery was placed on a paper to absorb the dissociated fat solution, and then the LNs were isolated from the dissociated mesentery by gastrointestinal surgeons. Both procedures were performed by eight different gastrointestinal surgeons randomly. All isolated LNs were then treated with standard formalin for fixation and hematoxylin and eosin for staining. Several pathologists evaluated the LNs for diagnosis.

### Fat-dissociation (FD) solution preparation

We previously reported the composition of FD solution as follows: 1 mg/ml collagenase (C6885; Sigma-Aldrich, St. Louis, MO, USA) and 0.25% trypsin (25200072; Life Technologies, Carlsbad, CA, USA) in Dulbecco’s phosphate-buffered saline (14190250; Life Technologies)^22^. Several additional conditions were examined as well (Supplementary Table S1). Finally, we modified the fat-dissociation solution as follows: 0.5 mg/ml collagenase (Sigma-Aldrich) and 5 mg/ml lipase (100817; MP Biomedicals, Santa Ana, CA, USA) in the normal saline buffer (Otsuka, Tokyo, Japan).

### Statistical analysis

Data were expressed by categories and/or median value (range) for continuous variables. The clinical and surgical factors were compared between the PM and the FM groups using the Wilcoxon rank-sum and χ^2^ test. Kaplan–Meier survival curves were plotted and prognosis was compared by the generalized log-rank test. All data were analyzed with JMP software (version 13.0; SAS Institute, Cary, NC, USA). Differences with a *P*-value of < 0.05 were considered statistically significant.

## Results

**Figure 2:**
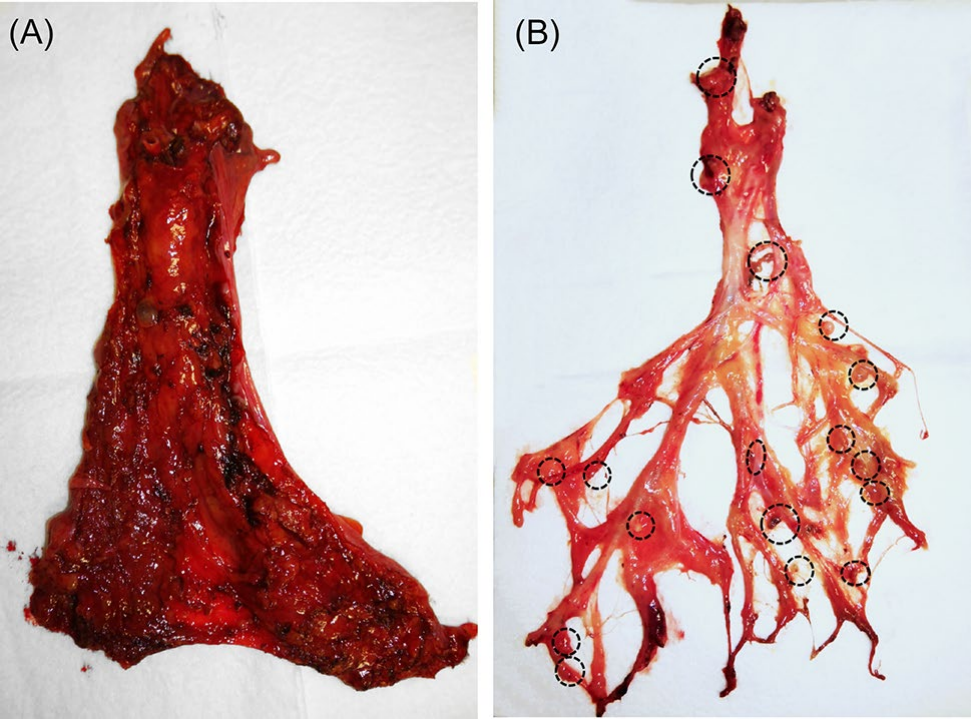
Surgically resected mesentery before and after the fat-dissociation procedure. (**A**) The mesentery was carefully separated from the primary tumor lesion. (**B**) Vessel structures and lymph nodes (circled) were visualized after the fat-dissociation method was applied.

In total, 130 patients were registered and assessed between January and December 2014. The number of patients exceeded the planned sample size. Overall, 65 patients were included in the PM group, and another 65 patients were included in the FM group. No patient dropped out from this study for evaluation of the primary endpoint. There were 35 (54%) and 36 (55%) male patients in the PM and the FM groups, respectively. The median BMI was 21.0 (range 14.4–29.8) and 21.0 (range 14.6–28.3) kg/m^2^ in the PM and the FM groups, respectively. LN dissection was D2 (51%) and D3 (49%) in the PM group and D2 (48%) and D3 (52%) in the FM group. There were no significant differences in the patients’ characteristics between the two groups (Table 1). The mesentery after FM is shown in Fig. 2. Most fat was dissociated, and the vascular structure together with LNs were clearly visible. LNs were located around vessels, and the LN region was easily identified. The median searching time was 45 min (range 15–80) in the PM group and 15 min (range 7–30) in the FM group. The time required for LN identification was significantly shorter in the FM group than in the PM group (*P* < 0.001). The median number of detected LNs that could be examined pathologically was 16 (range 2–48) in the PM group and 18 (range 4–95) in the FM group. The number of LN did not differ significantly between the two groups (*P* = 0.546). These results were summarized in Table 2.

**Table 1:** Patients’ characteristics.

Factors	Palpation method group (n = 65)	Fat-dissociation method group (n = 65)	*P* value
Age^a^	66 (33–90)	66 (31–86)	0.808
Sex (male/female)	35/30	36/29	0.860
BMI (kg/m^2^)^a^	21.0 (14.4–29.8)	21.0 (14.6–28.3)	0.570
Tumor location: (right side/left side)	41/24	43/22	0.714
Procedure: (ICR/R/T/L/S/AR/LAR)	4/18/4/4/18/8/9	6/14/2/6/18/10/9	–
Lymph node dissection^b^ (D2/D3)	33/32	31/34	–
Tumor invasion (T1-2/T3-4)	28/37	29/36	0.860
Lymph node metastasis: (absent/present)	42/23	34/31	0.154
Stage^c^ (0/I/II/III/IV)	0/26/15/21/3	1/23/11/24/6	–

Right side; cecum, ascending colon and right-side transverse colon, Left side; left-side transverse coclon, descending colon, sigmoid colon, and rectum.

*ICR* ileocecal resection, *R* right colectomy, *T* transverse colectomy, *L* left colectomy, *S* sigmoid colectomy, *AR* anterior resection, *LAR* low anterior resection.

^a^Continuous variables; median (range).

^b^Lymph node dissection was decided according to Japanese clinical guidelines, Japanese Classification of Colorectal Carcinoma.

^c^Stage was decided according to Japanese clinical guidelines, Japanese Classification of Colorectal Carcinoma.

**Table 2:** Results of searching time and number of lymph nodes.

	Palpation method group (n = 65)	Fat-dissociation method group (n = 65)	*P* value
Searching time	45 (15–80)	15 (7–30)	< 0.001
Number of lymph nodes	16 (2–48)	18 (4–95)	0.546
Number of metastatic lymph nodes	0 (0–25)	0 (0–25)	0.157

**Figure 3:**
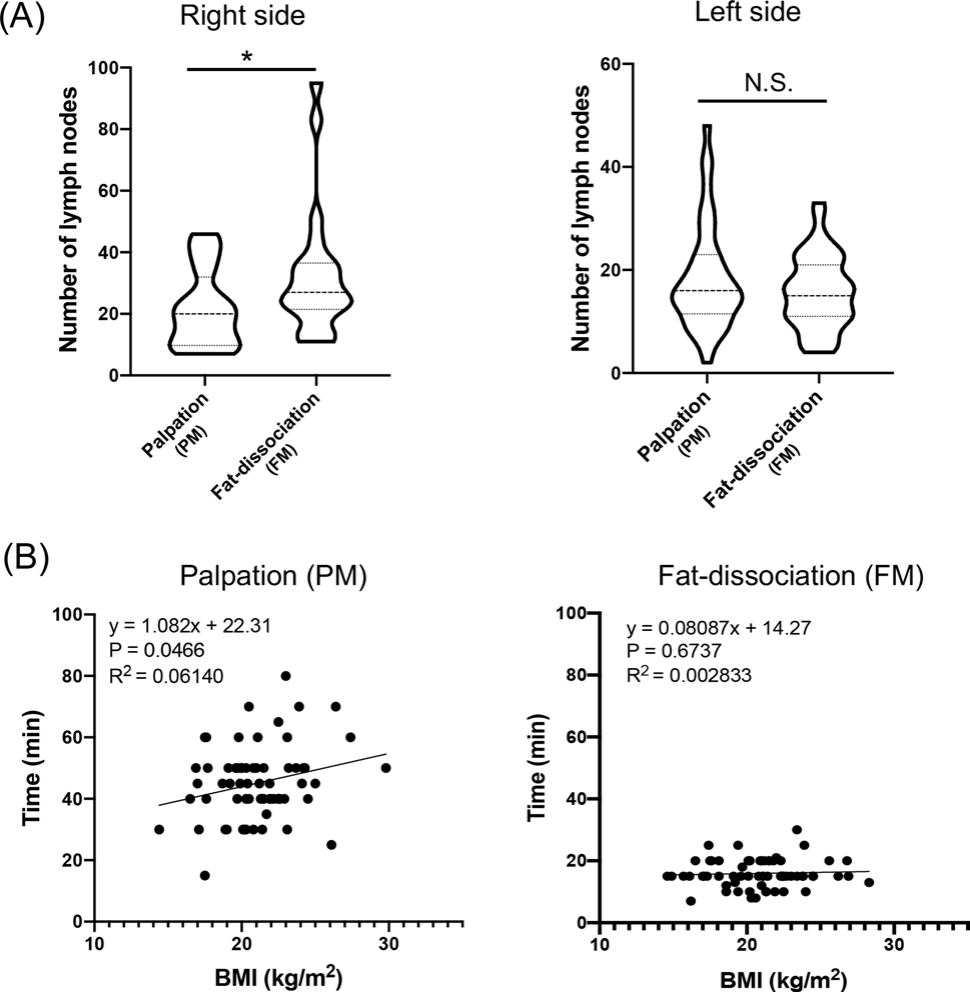
Lymph node quantification according to tumor location and the relationship between LN identification time and BMI. (**A**) In right-side colorectal cancer, the median number of lymph nodes was 20 (range 7–46) in the PM group and 27 (range 11–95) in the FM group (*P = 0.031). In left-side colorectal cancer, the median number of lymph nodes was 16 (range 2–48) in the PM group and 15 (range 4–33) in the FM group (*P* = 0.408). (**B**) LN identification time increased with BMI in the PM group (R^2^ = 0.061), but there was no correlation in the FM group (R^2^ = 0.003).

**Figure 4:**
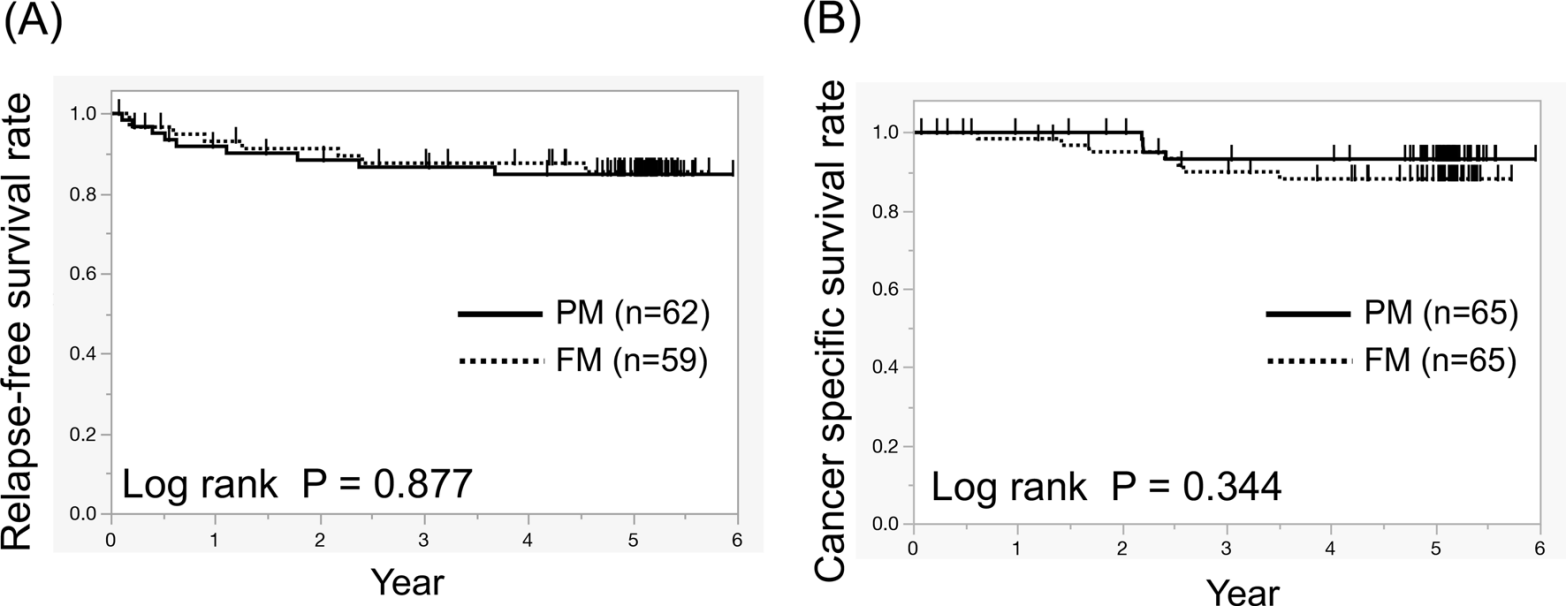
Relapse-free survival curves and cancer-specific survival curves. (**A**) 5-year postoperative relapse-free survival rate, except in those with stage IV disease was 85% in the PM group and 85% in the FM group. (**B**) 5-year postoperative cancer-specific survival rate in all patients was 93% in the PM group and 88% in the FM group.

For right-sided CRC, the number of LNs was higher in the FM group than in the PM group (*P* = 0.031), but the number of LNs did not differ significantly between the two groups for left-sided CRC (*P* = 0.407) (Fig. 3A). Moreover, the LN identification time correlated with BMI in the PM group (R^2^ = 0.061, *P* = 0.047), while it did not correlate with BMI in the FM group (R^2^ = 0.003, *P* = 0.674) (Fig. 3B). These results suggest that it is more difficult to identify LNs in fat-enriched rich specimens. Lastly, prognosis was examined. Postoperative RFS was examined in patients without stage IV. 5-year RFS rate was 85% in the PM group and 85% in the FM group. They did not differ between the two groups (*P* = 0.877) (Fig. 4). Postoperative CSS was examined in all patients. 5-year CSS rate was 93% in the PM group and 88% in the FM group. They also did not differ between the two groups (*P* = 0.344).

Additionally, LNs were examined pathologically, and there was no difficulty in diagnosing cancer after applying FM (Supplementary Fig. S1A). Protein expression for molecules such as epithelial cell adhesion molecule and CK20 could also be detected in LNs with metastasis. mRNA presence, that is more susceptible to degradation as compared with DNA, was also examined. *KRT19* mRNA expression was significantly higher in samples with metastasis even after FM (Supplementary Fig. S1B).

## Discussion

In cancer, proper LN detection is crucial for staging. However, the current methods for LN detection are time consuming and rely on extensive experience of the surgeons and pathologist. In this study, we examined the clinical usefulness of FM with respect to LN detection time and the number of LNs in CRC. The results showed that by dissociating fat tissue, FM improved clearing of the mesenteric structure, therefore making the LNs visible. FM allowed easier identification of LNs around vessels, thus reducing the time required for LN detection. In addition, a higher number of LNs were identified in samples obtained from right-sided CRC. Identifying LNs directly from surgically resected specimen is technically challenging because the color of LNs is similar to that of fat. Such identification mostly depends on the experience of the researcher. In the cases of right-sided CRC, the range of mesenteric size is large, and identification took time. Therefore, if the amount of lymph nodes searched is wrongly identified to be sufficient, it may result in downstaging because of the lower number of lymph nodes detected. The fat-dissociation method that we developed can be used even by inexperienced researchers as it will enable to easily recognize LNs, and it seems to be a useful method for direct procedures such as OSNA^18^ which requires LNs freshly isolated from surgically resected specimens. In our protocol for preparation of the fat-dissociation solution, the incubation time was set from 30 to 60 min for safety reasons. However, if a higher concentration solution is used, the incubation time could be shorter. Therefore, high concentrations and short periods of time may be desirable, such as when searching for sentinel lymph nodes for OSNA during surgery. In this study, we set the low concentration for safety. Because if we missed the time, the LN will be damaged in a high-concentration solution. However, if there is a dedicated person performing the work, the time will be shorter with higher concentration. The concentration of this solution could be modified and would be applicable in several institutions in several situations. In addition, our fat-dissociation solution at low concentration was commercialized as Imofully (Sysmex, Kobe, Japan). Similar to our study, it was reported that the results were excellent in short-term results such as the number of lymph nodes and the search time by using Imofully (Sysmex)^24,25^.

It has been reported recently that molecular characterization of cancer based on gene expression is important for proper disease stratification^26^, and the appropriate drug treatment is selected according to specific mutations^2,9^. We examined the *KRT19* mRNA expression via RT-PCR analysis. The *KRT19* mRNA levels serve as useful marker to detect the metastasis of CRC to the LN^27^. Our results suggested that FM did not interfere with the detection of mRNA expression and would be a useful pre-method in the diagnosis of LN metastasis using RT-PCR system such as OSNA in the future. Furthermore, it may be possible to automate the LN diagnosis by combining FM with the OSNA method.

However, this study has several limitations. One is that this is a single-center study. Second, the study needs to be verified not only in surgeons, but also in other personnel including laboratory technicians. Moreover, the primary endpoint was not prognostic in this study. We did not specify the tumor stage in this study; there were more patients with advanced stage IIIb and IV in the FM group. Focusing on stage II and IIIa, where more accurate lymph node identification is reflected in the stage, the 5-year RFS was 77% and 87% and the 5-year CSS was 93% and 100% in the PM and the FM groups, respectively. However, the difference was not significant, and additional study is required to verify the effect of FM on prognosis. In addition, postoperative adjuvant chemotherapy was not specified, and the number of cases was not sufficient for prognostic analysis. Therefore, we could not mention that FM was an essential method to determine the N factor that reflect prognosis. More patients with stage II–III need to be evaluated to determine whether FM is a truly useful method for determining the N factor. Nevertheless, FM can help visualize LNs in surgical specimens and reduce the time required for identification and diagnosis of LNs in cancer tissue.

In conclusion, FM reduced the time required for LN identification by allowing better and more efficient visualization of LNs in surgically resected mesentery. This is an innovative useful method to detect LNs from surgical specimens.

## Supplementary information

Supplementary Information.

## Data Availability

All data of our results was included.

## Abbreviations

CRC: Colorectal cancer
LN: Lymph nodes
FM: Fat-dissociation method
PM: Palpation method
OSNA: One-step nucleic acid amplification
OICI: Osaka International Cancer Institute
KRT19: Cytokeratin 19
CK20: Cytokeratin 20
GAPDH: Glyceraldehyde-3-phosphate dehydrogenase
RT-PCR: Real-time polymerase chain reaction
