# Supplementary material for: A new fat-dissociation method to detect lymph nodes in colorectal cancer: a prospective randomized study
Source: Sci Rep. 2020 Nov 19;10:20205. doi: 10.1038/s41598-020-77195-8 (PMC7678840; doi:10.1038/s41598-020-77195-8)
Supplement: Supplementary file 1 — Supplementary Information. [file 41598_2020_77195_MOESM1_ESM.docx]

Supplementary Information for

**Original Article**

**Title: A new fat-dissociation method to detect lymph nodes in colorectal cancer: A prospective randomized study**

Shiki Fujino^1,2^, Norikatsu Miyoshi^1,2^, Masayuki Ohue^3^, Aya Ito^1^, Masayoshi Yasui^3^, Takayuki Ogino^2^, Hidekazu Takahashi^2^, Mamoru Uemura^2^, Chu Matsuda^2^, Hirofumi Yamamoto^2^, Tsunekazu Mizushima^2^, Yuichiro Doki^2^, Hidetoshi Eguchi^2^, Nariaki Matsuura^3^

^1^ Innovative Oncology Research and Translational Medicine, Osaka International Cancer Institute, 3-1-69, Otemae, Chuo-ku, Osaka 541-8567, Japan

^2^ Department of Gastroenterological Surgery, Osaka University Graduate School of Medicine, 2-2, Yamadaoka, Suita-City, Osaka 565-0871, Japan

^3^ Department of Surgery, Osaka International Cancer Institute, 3-1-69, Otemae, Chuo-ku, Osaka 541-8567, Japan

Corresponding author: Norikatsu Miyoshi

Department of Gastroenterological Surgery, Osaka University Graduate School of Medicine

2-2, Yamadaoka, Suita-City, Osaka 565-0871, Japan

E-mail: nmiyoshi@gesurg.med.osaka-u.ac.jp

**This file includes:**

Supplementary methods

Figure S1

Table S1

**Supplementary methods**

***RNA preparation and expression analysis***

Total RNA was extracted using the RNA Purification kit (Qiagen GmbH, Hilden, Germany). A Transcriptor First Strand cDNA Synthesis kit (Roche Diagnostics, Tokyo, Japan) was used to perform reverse transcription. Two human cytokeratin 19 (*KRT19)* oligonucleotide primers were designed for the polymerase chain reaction (PCR) as follows: forward 5'-AGGGTGCTGGATGAGCTG-3' and reverse 5'-CCCCTCAGCGTACTGATTTC-3'. KRT19 expression was normalized by another gene expression, the glyceraldehyde-3-phosphate dehydrogenase (GAPDH) gene. Two human GAPDH oligonucleotide primer sequences were as follows: forward 5'-AGC CACATCGCTCAGACAC-3' and reverse 5'-GCCCAATAC GACCAAATCC-3'. As positive controls, RNA extracted from NTERA-2 cancer cells were concurrently studied. Quantitative assessments were performed by real-time reverse transcription-PCR (RT-PCR) using a Universal ProbeLibrary platform (Roche Diagnostics. #80 for *KRT19,* and #60 for *GAPDH*) and FASTStart TaqMan Probe Master (Roche Diagnostics) for cDNA amplification of the target genes. The expression of *KRT19* mRNA were calculated after normalization using *GAPDH* mRNA expression.

***Immunohistochemistry***

Formalin-fixed, paraffin-embedded LNs were used. After deparaffinization and blocking, the sections were incubated with primary anti-E Cadherin mouse monoclonal antibody (ab1416; Abcam, Cambridge, MA) at the dilution of 1:50 and anti-cytokeratin 20 (CK20) mouse monoclonal antibody (ab854; Abcam) at the dilution of 1:50 overnight at 4°C. The signal was detected using Vectastain Universal Elite kit (Vector Laboratories, Burlingame, CA, USA). Diaminobenzidine was used for color modification. All sections were counterstained with hematoxylin.

**Figure S1. Analysis of detected lymph nodes**


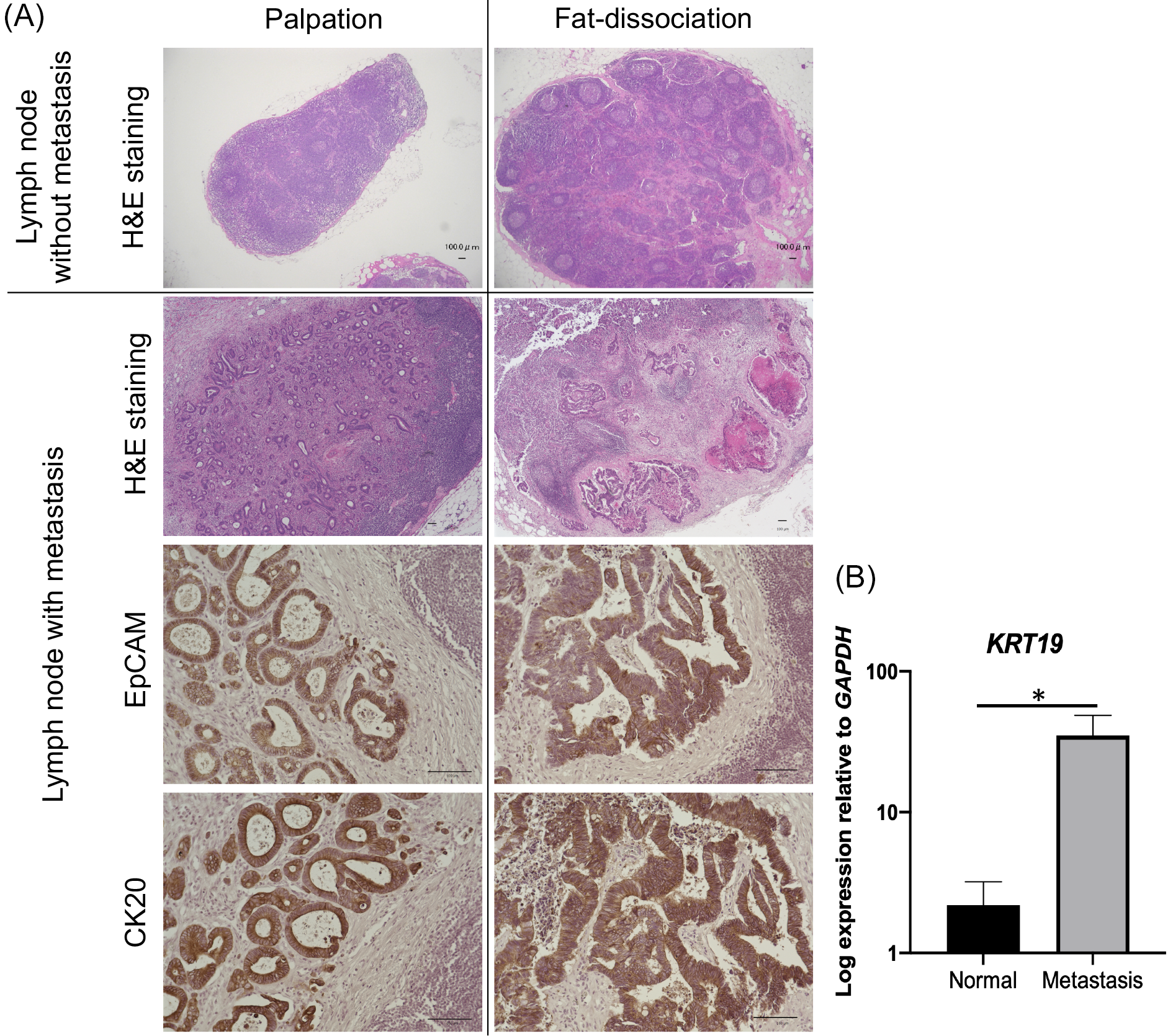


(A) Representative images of hematoxylin and eosin (H&E) staining and immunohistochemistry of lymph node without metastasis, and with metastasis in both methods. Metastasis was diagnosed via H&E staining. Protein expression of epithelial cell adhesion molecule and CK20 was detected in lymph nodes using both methods. Scale bar: 100 μm. (B) *KRT19* mRNA expression in the lymph nodes detected using the fat-dissociation method. It was significantly higher in lymph nodes with metastasis than in normal lymph nodes. (**P*<0.05)

**Table S1. Conditions of the fat-dissociation method.**

| **Collagenase**  **(mg/ml)** | **Trypsin**  **(%)** | **Lipase**  **(mg/ml)** | **Incubation time** | **Temperature** | **Dissociation*** |
| --- | --- | --- | --- | --- | --- |
| 0 | 0.25 |  | 120 | 40 | 2 |
| 0.5 | 0.05 |  | 30 | 22 | 1 |
| 0.5 | 0.125 |  | 45 | 37 | 3 |
| 0.5 | 0.125 |  | 7200 (12hrs) | 22 | 3 |
| 1 | 0 |  | 30 | 22 | 2 |
| 1 | 0.25 |  | 360 | 22 | 3 |
| 1 | 0.25 |  | 15 | 37 | 3 |
| 1 | 0.25 |  | 10 | 40 | 3 |
| 0.5 | 0.125 | 5 | 10 | 40 | 2 |
| 0.5 | 0.125 | 5 | 30 | 40 | 3 |
| 1 | 0.25 | 10 | 10 | 40 | 3 |
| 0.5 |  | 5 | 10 | 40 | 2 |
| 0.5 |  | 5 | 30 | 40 | 3 |
| 0.5 |  | 10 | 10 | 40 | 2 |
| 0.5 |  | 10 | 30 | 40 | 3 |
| 1 |  | 10 | 10 | 40 | 2 |
| 1 |  | 10 | 30 | 40 | 3 |

* Estimation of dissociation: 1, weak; 2, moderate; 3, good.
